# *De novo* peroxisome biogenesis revisited

**DOI:** 10.15698/mic2014.04.138

**Published:** 2014-04-01

**Authors:** Marten Veenhuis, Ida J. v. d. Klei

**Affiliations:** 1 Molecular Cell Biology, Groningen Biomolecular Sciences and Biotechnology Institute, University of Groningen, The Netherlands.

**Keywords:** yeast, peroxisomes, de novo peroxisome formation, peroxisome deficient mutant, endoplasmic reticulum

## Abstract

We describe an alternative peroxisome formation pathway in yeast *pex3* and *pex19 *cells, which relies on the existence of small peroxisomal remnants that are present in these cells. This groundbreaking result challenges current models prescribing that peroxisomes derive *de novo* from the ER. Our data also has major implications for the sorting pathway of specific peroxisomal membrane proteins (PMPs). We propose a novel sorting pathway for the PMPs Pex13 and Pex14 that is independent of the known Pex3/Pex19 machinery.

Peroxisomes are crucial, multifunctional organelles the abundance and function of which continuously adapt to satisfy cellular needs. The development of these organelles is strongly debated. Current models differ from multiplication by fission via dynamin-related protein (Drp) dependent fission machineries, which are well documented now. An alternative model prescribes that most, if not all, organelles form *de novo* from the endoplasmic reticulum (ER). *De novo* peroxisome biogenesis is most studied following functional complementation of *PEX3* deletion (*pex3*) stains, which so far were assumed to fully lack peroxisomal membrane structures. Fluorescence microscopy (FM) analysis of complemented cells revealed that newly synthesized Pex3-GFP sorts to the ER, concentrates in foci followed by the formation of a pre-peroxisomal structure, which pinches off and develops into a nascent peroxisome. Alternatively, two (in *Saccharomyces cerevisiae*) or multiple (in *Yarrowia lipolytica*) types of vesicles have been proposed developing from the ER which subsequently fuse to form a nascent peroxisome.

We have now shown that these models are no longer generally valid as *Hansenula polymorpha* and *S. cerevisiae* *pex3* cells, other than generally anticipated, do contain small peroxisomal membrane remnants (ghosts), which are the target for reintroduced Pex3 and the template for subsequent peroxisome formation. Similar observations were made in *pex19* cells of both species although the *pex19 *vesicles differed from those present in *pex3* cells in that they contained, besides Pex13, Pex14 and Pex8, also Pex3. Pex13 and Pex14 are key components of the matrix protein receptor docking complex. In the membrane remnants small amounts of matrix protein were present suggesting that Pex13 and Pex14 were correctly inserted and functional as receptor docking site. The low matrix content may be explained in that the proteins of the receptor recycling system (including the RING finger proteins) were not present on these structures, thereby preventing recycling of the PTS1 receptor Pex5. Indeed, Pex5 was found associated with the vesicles, whereas other PMPs, e.g. PMP47 and the RING finger proteins, were unstable and present in low amounts in the cytosol.

These findings have major implications for the current concepts of peroxisome *de novo* formation. These models invariably prescribe that the ER is the membrane template for *de novo* synthesis of which we showed that this is no longer generally valid. Most likely, this can be explained by the relatively low resolution of the fluorescence microscopy (FM) techniques used. Also, in most cases Pex3 synthesis was driven by the strong, inducible galactose promoter giving rise to relative high Pex3 protein levels at the initial stages of peroxisome re-induction compared to the low levels produced under control of the endogenous *PEX3* promoter. This initial overexpression effect most likely resulted in mislocalization of excess Pex3-GFP at the ER. As the vesicular structures in *pex3* and *pex19* cells are generally localized in close vicinity of the ER, the resolution of FM is insufficient for discriminating the vesicular structures from the ER. The latter could only be achieved with the high resolution microscopy techniques we applied. Possibly, the Pex3 puncta that have been described before to be localized at the ER at the initial stage of pre-peroxisome formation in fact represent the vesicular structures. Using Pex3 synthesis driven under the control of the endogenous *PEX3* promoter we have never seen localization of Pex3 to the ER but did so when synthesis was under control of the strong alcohol oxidase promoter.

**Figure 1:**
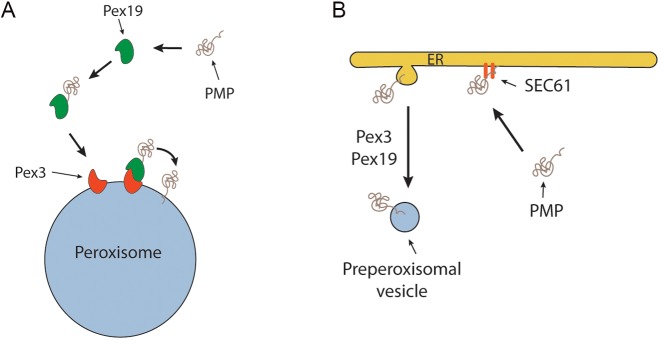
FIGURE 1: Current PMP sorting models. **(A)** PMP sorting requires the Pex3/Pex9 sorting machinery. In this classical model Pex19 functions as receptor/chaperone to sort newly synthesized PMP molecules to the peroxisomal docking site Pex3 followed by PMP insertion in the membrane by yet unknown mechanisms. **(B)** PMP sorting requires the ER. According to this model peroxisomes are formed from the ER. PMPs are first inserted in the ER dependent of the Sec61 machinery and subsequently incorporated in single or multiple vesicles that are separated from the ER via the function of Pex3 and Pex19. In case of multiple vesicles, these fuse to form a pre-peroxisomal vesicle.

Together, our observations convincingly show that the ER is not the initial membrane template for peroxisome formation in *pex3* and *pex19* mutants and hence that upon complementation of these mutants with the corresponding genes the organelles are formed from a pre-peroxisomal structure and not *de novo* (i.e. from a template unrelated to peroxisomes). It also uncovered a novel mechanism of PMP sorting. Towards this, so far two main models existed, explaining the absence of peroxisomal membrane structures in *pex3 *and *pex19* cell (Fig. 1). One of these proposes that, upon synthesis in the cytosol on free ribosomes, PMPs are sorted to the target organelle via the Pex19/Pex3 sorting machinery (Fig. 1A). In this machinery Pex19 functions as receptor/chaperone to bind newly synthesized PMPs and transports them to Pex3, serving as membrane docking site. The second model (Fig. 1B) prescribes that all PMPs travel via the ER, from which pre-peroxisomal vesicles are formed. In this model Pex3 and Pex19 are required for exit of the PMPs from the ER in vesicles. Together, our novel data provide new conceptual insight into peroxisome biogenesis. First, it changes the concept of *de novo* peroxisome formation from the ER, as this machinery most likely does not exist. This does not imply that the ER may not play a role at all, but most likely solely serves a function in membrane lipid supply. Also a role in the formation in the vesicular structures present in *pex3* and *pex19* cells cannot be excluded (Fig. 2), but our data clearly do not fit with a model that all peroxisomes arise from the ER. Possibly, peroxisome inheritance mutants are favorite models to study *de novo* peroxisome biogenesis provided that these cells do not contain the vesicular structures observed in *pex3* and *pex19 *mutants. Our data confirm that most PMPs, which are unstable and soluble in the mutant cells, rapidly stabilize and sort to the vesicular structures in a Pex19/Pex3 dependent manner upon functional complementation of the corresponding *pex3 *or *pex19* mutant (Fig. 2). Hence, these are not sorted via the ER. Furthermore, in *pex3* and *pex19* cells the vesicular membrane structures contained Pex13, Pex14 and Pex8 indicating that these proteins reach these structures independent of Pex3 and Pex19. How these proteins reach their target membrane is yet fully unclear but possibly may involve the function of the ER (Fig.2). Also, we have never seen Pex3 accumulating in the ER when produced from its own promoter. However, the residence time of this protein in the ER may be too short to track its routing via FM. Therefore, routing of Pex3 requires further investigation using advanced microscopy and biochemical techniques.

**Figure 2:**
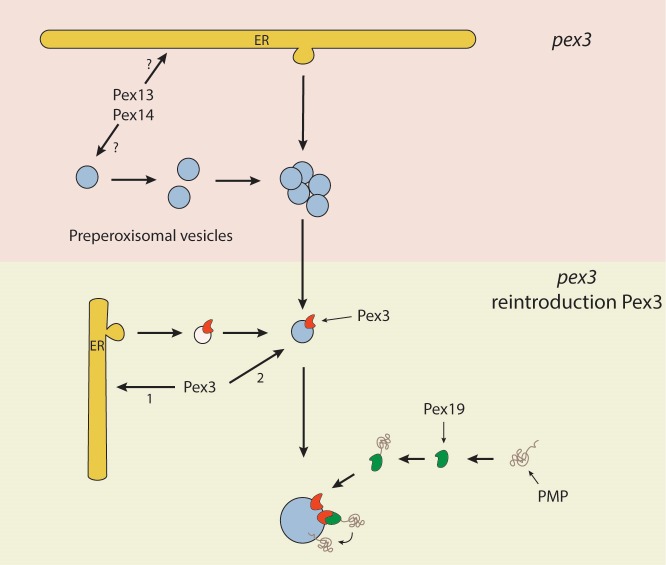
FIGURE 2: Schematic overview of the novel peroxisome biogenesis pathway. The upper pink part represents the presence of pre-peroxisomal vesicles, containing Pex13 and Pex14, in yeast *PEX3* deletion cells. These vesicles may be autonomous and proliferate by fission or form from the ER in a Pex3/Pex19 independent manner. Upon re-introduction of Pex3 (lower part in green), the protein most likely directly travels to the pre-peroxisomal vesicles or alternatively, reaches these via ER derived vesicles. After incorporation of Pex3 in the pre-peroxisomal vesicles, the other PMPs insert into the vesicles via the Pex3/Pex19 dependent docking machinery (see also Fig. 1A).

The present work has opened new avenues to unravel the principles of peroxisome biogenesis. Many questions remain. An urgent question to solve is on the origin of the vesicular structures in *pex3* and *pex19* cells. Are they autonomous or do they derive from the ER? What are the protein components essential for their formation? Yet, the first component has been identified in two independent studies. We and the Hartig group identified Pex25 as being essential for *de novo* peroxisome formation in yeast *pex3* cells and in young buds of *inp2* cells that lacked peroxisomes through an inheritance defect.

